# Supplementary material for: The microbiome of the buffalo digestive tract
Source: Nat Commun. 2022 Feb 10;13:823. doi: 10.1038/s41467-022-28402-9 (PMC8831627; doi:10.1038/s41467-022-28402-9)
Supplement: Supplementary file 1 — Supplementary information [file 41467_2022_28402_MOESM1_ESM.pdf]

Supplementary information

Supplementary Table 1 Numbers of samples from different sampling areas.

Supplementary Table 2 Numbers of samples from different breeds.

Supplementary Table 3 Numbers of samples from different growth phases.

Supplementary Table 4 Numbers of samples from different sources.

Supplementary Fig. 1 Methodological overview of our metagenomic analysis workflow.

Supplementary Fig. 2 Quality assessment and taxonomic classification of the 3,255 species-level MAGs.

Supplementary Fig. 3 Comparison of the genome size and completeness of our MAGs with public datasets.

Supplementary Fig. 4 Mapping rates of the reads from Stewart et al. to our MAGs and cattle RUGs.

Supplementary Fig. 5 Taxonomic assignments of 4,960 MAGs at different taxonomic levels.

Supplementary Fig. 6 Comparisons of the alpha diversity of DT-associated microbes among different digestive tract (DT) sections and sites.

Supplementary Fig. 7 Principal coordinates analysis (PCoA) of the Bray-Curtis distances of DT-associated microbes among different digestive tract (DT) sections and sites.

Supplementary Fig. 8 Boxplot of the Prevotella species with the highest abundance in the rumen.

Supplementary Fig. 9 Correlation analysis among genera with known taxonomic classifications using their relative abundance in all DT samples.

Supplementary Fig. 10 Relative abundance of all marker taxa in different sections along the digestive tract.

Supplementary Fig. 11 Maximum percentage identity between predicted proteins from the MAGs and the CAZy database.

Supplementary Fig. 12 Comparison of the relative abundance of six CAZy families between buffalo and cattle based on all CAZy protein abundance values.

Supplementary Fig. 13 Effects of different assembly parameters on read mapping rates against the obtained scaffolds (left panel) and bins (right panel).

Supplementary Fig. 14 Quality assessment of the nonredundant MAGs generated using the Kmer_MRate and Kmer_N50 methods.

Supplementary Fig. 15 Comparison of the assembly results between metaSPAdes and MEGAHIT.

Location		Sample number
**China**	Guangxi	471
	Henan	41
	Anhui	44
	Yunnan	93
	Hainan	31
	Hubei	15

Supplementary Table 1 Numbers of samples from different sampling areas.

	Breed	Sample number
**River buffalo**	Mediterranean Buffalo	55
	Guangxi river buffalo	12
	Nili-Ravi buffalo breeding stock	18
	Murrah buffalo	22
	Penang river buffalo	31
	Italy river buffalo	15
**Swamp buffalo**	Guangxi swamp buffalo	252
	Xinyang swamp buffalo	41
	Anhui swamp buffalo	44
	Myanmar swamp buffalo	31
	De Hong swamp buffalo	31
	Hainan swamp buffalo	31
**Hybrid buffalo**	——	112

Supplementary Table 2 Numbers of samples from different breeds.

Growth phases	Sample number
**Adult Buffalos**	659
**Calf**	36

Supplementary Table 3 Numbers of samples from different growth phases.

Source		Sample number
	Rumen	129
	Reticulum	28
	Omasum	32
**Contents**	Abomasum	22
	Jejunum	24
	Caecum	31
	Colon	30
**Faeces**	Rectum	399

Supplementary Table 4 Numbers of samples from different sources.

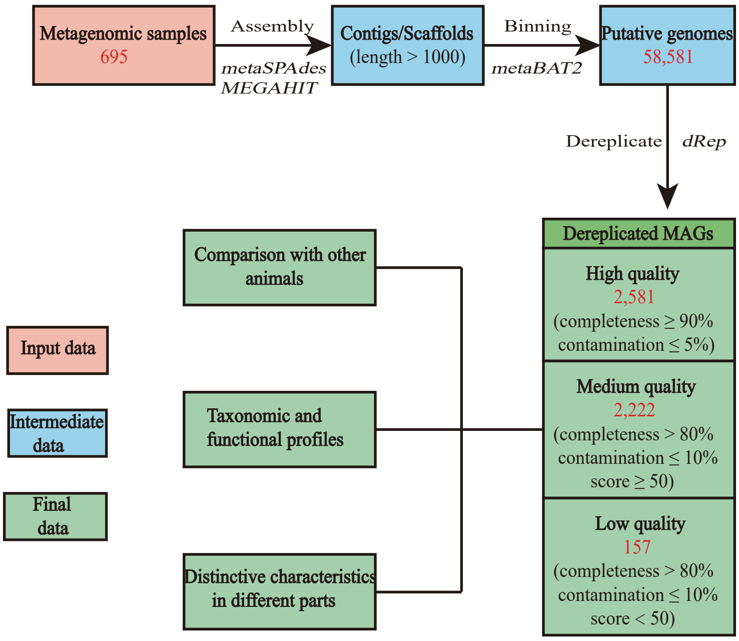

Supplementary Fig. 1: Methodological overview of metagenomic analysis. The flow diagram of metagenomic analysis.

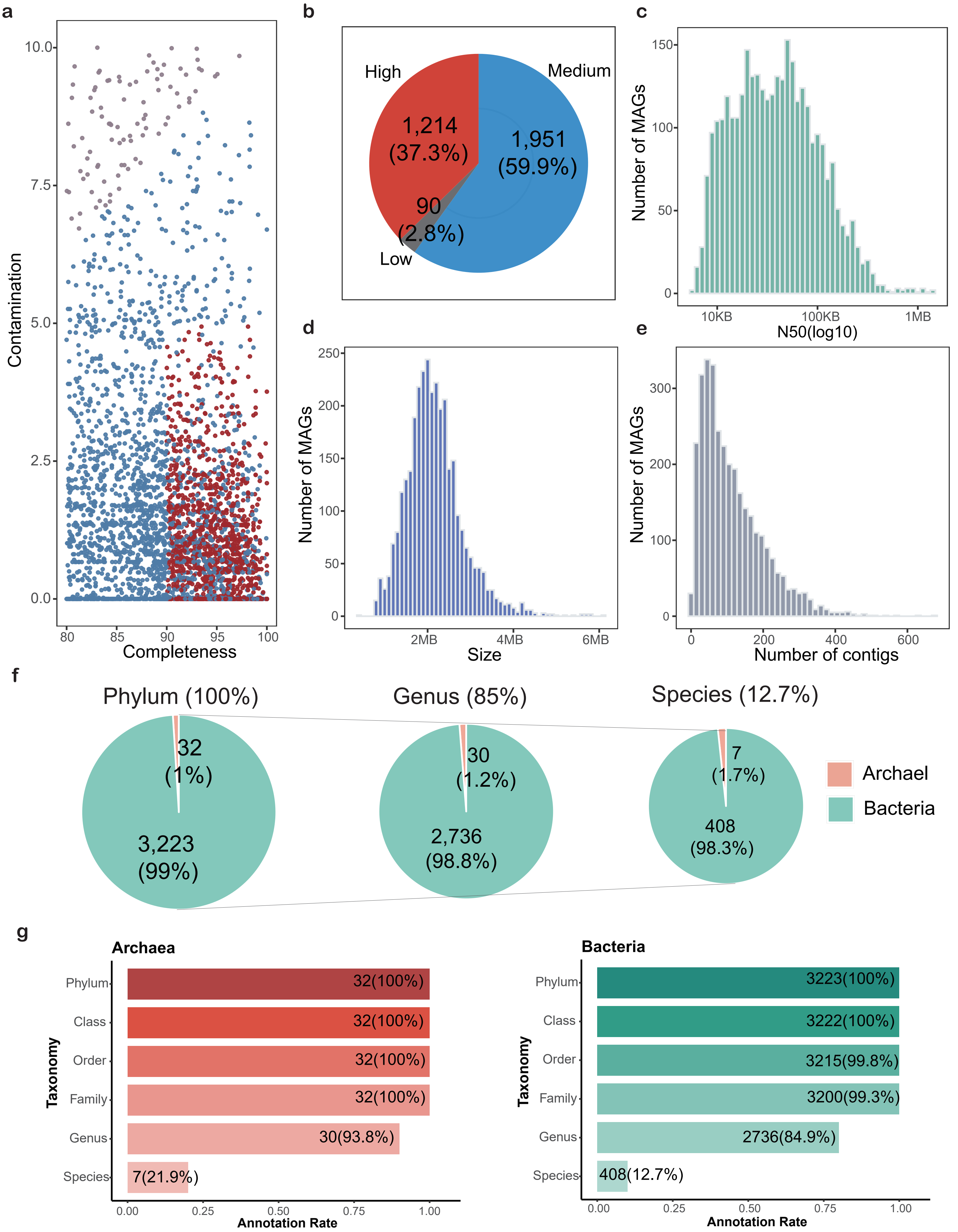

Supplementary Fig. a2: Quality assessment and Classification of 3,255 species-level MAGs. a) Each point represents a MAG. Red points indicate the highest quality genomes with ≥90% completeness, ≤5% contamination, and presence of the 23S, 16S, and 5S rRNA genes and at least 18 tRNAs. All other MAGs are >80% complete and ≤10% contaminated. Those in blue have a quality score ≥50, whereas those in grey have a quality score <50. b) Pie chart shows the numbers and relative proportions of the red, blue, and grey MAGs in c). Histograms in c), d) and e) show the distributions of N50, genome size, and the number of contig per genome respectively for the 3,255 MAGs. f) The classification rates of 3,255 MAGs at different taxonomic levels. The numbers above the pie charts indicate the percentages of MAGs (out of 3,255) that could be annotated at the respective levels; the numbers inside the pie charts indicate the percentages of archaea (orange) and bacteria (green) of each pie. g) The classification rates of archaea (left) and bacteria (right) at different taxonomic levels. The numbers indicate the amounts of MAGs classified.

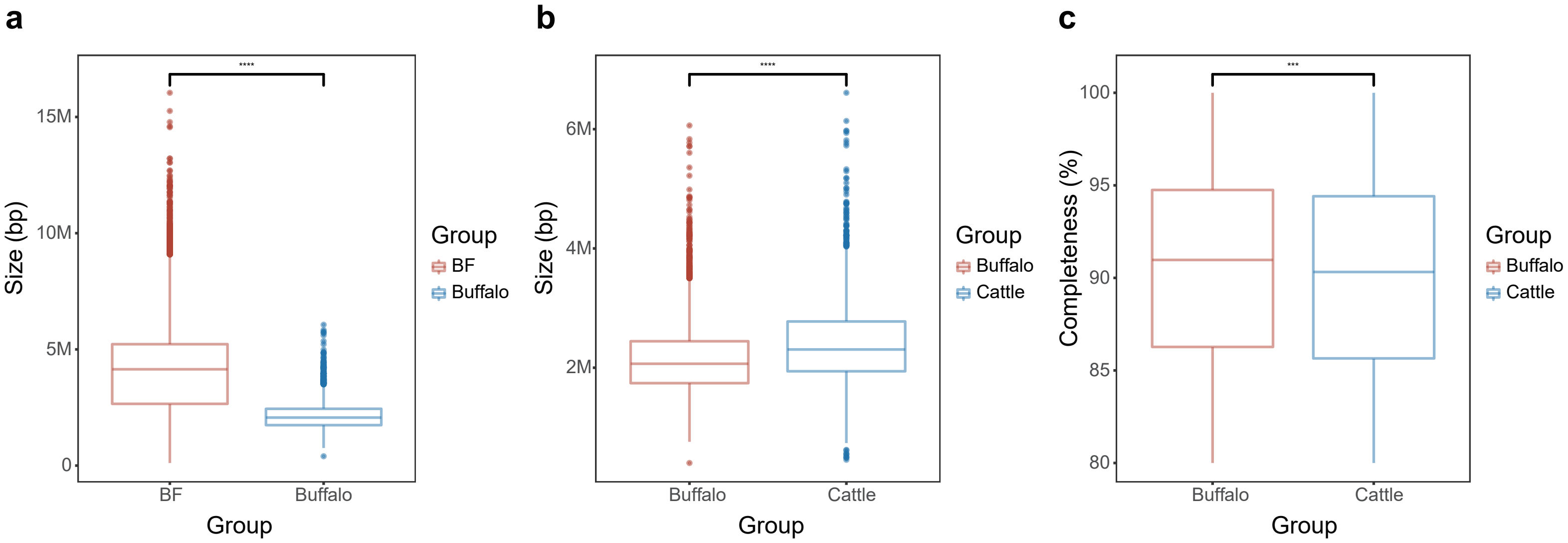

Supplementary Fig. 3: Boxplot comparison of the genome size and genomes completeness of three datasets. Comparison of genome size between Buffalo strain-level MAGs and a) NCBI Refseq prokaryotic genomes, b) Cattle MAGs. c) Comparison of completeness between Buffalo strain-level MAGs and Cattle MAGs. BF is the the NCBI Refseq prokaryotic genomes (457 archaeal and 28,011 bacterial genomes with assembly levels of complete or chromosome (downloaded as of June 28, 2021)). Cattle is the 4,941 cattle rumen MAGs from Stewart et al. Boxplots show median, 25th and 75th percentile, the solid line indicate the minima and maxima, and the points laying outside the whiskers of boxplots represent the outliers. Pair-wise Wilcoxon Rank Sum Test was used to compare between the groups.
Level of significance: ns P≥ 0.05, * P < 0.05, ** P < 0.01, *** P < 0.001, **** P <0.0001.

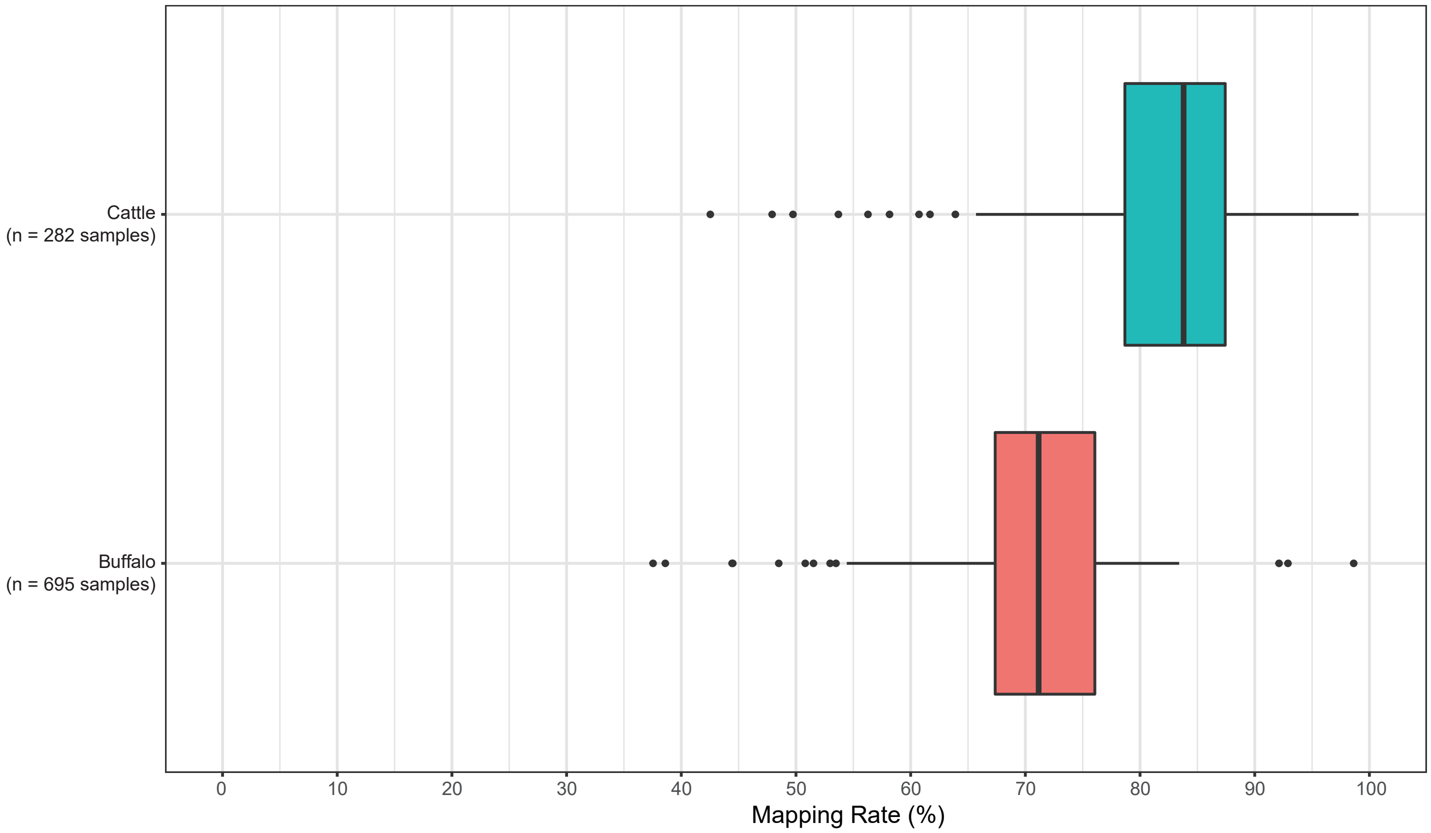

Supplementary Fig. 4: Mapping rate for our strain-level MAGs and RUGs against the reads of Stewart et al. Red box and cyan box indicate the percentages of metagenomics reads from Stewart et al with MAGs of buffalo (n = 695 samples) and RUGs of cattle (n = 282 samples), respectively. Boxplots show median, 25th and 75th percentile, the solid line indicate the minima and maxima, and the points laying outside the whiskers of boxplots represent the outliers.

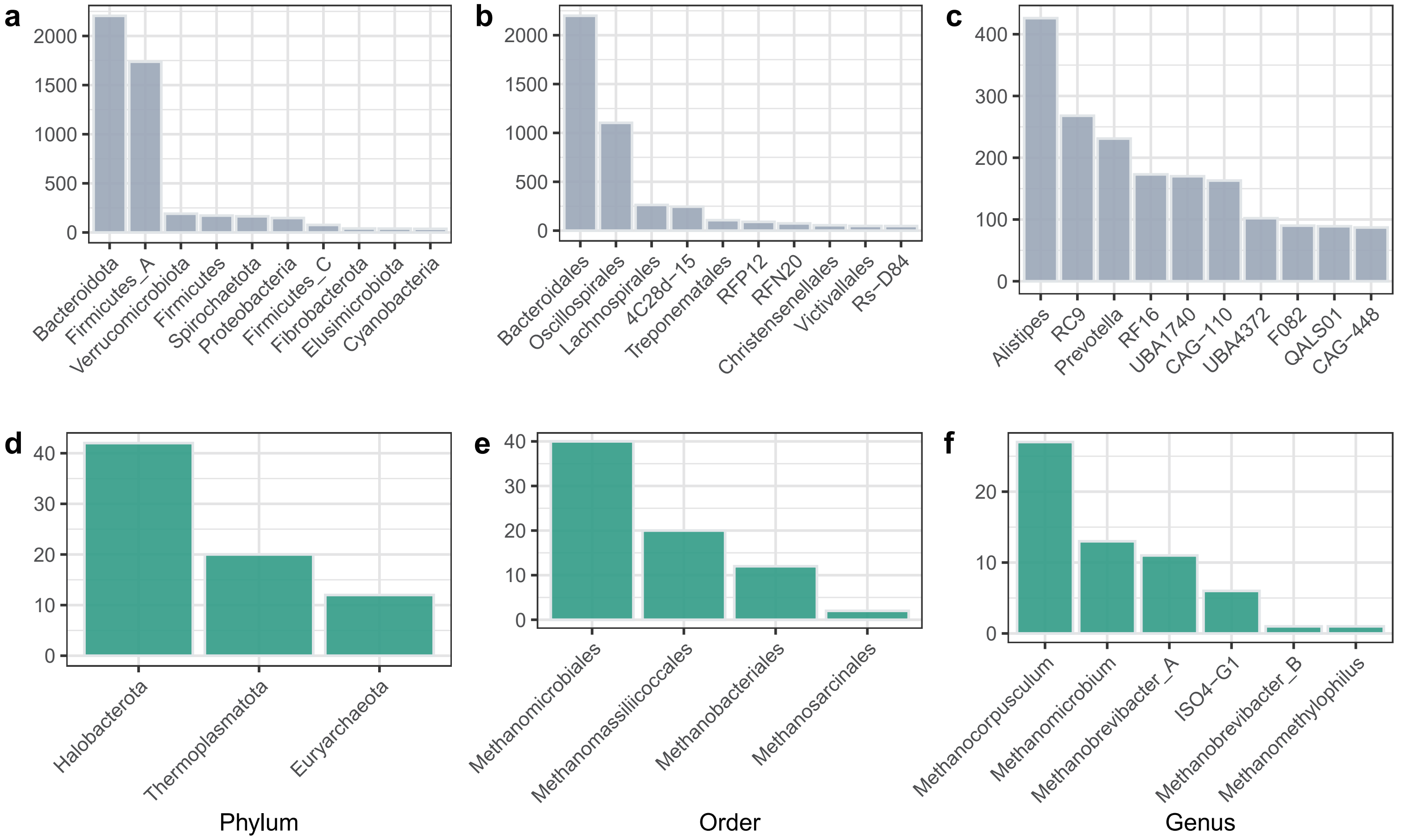

Supplementary Fig. 5: Taxonomic assignments of 4,960 strain-level MAGs. X-axis indicate bacteria and archaea classified to phylum (a, d), order (b, e), and genus (c, f) level, respectively. Y-axis indicate the number of MAGs.

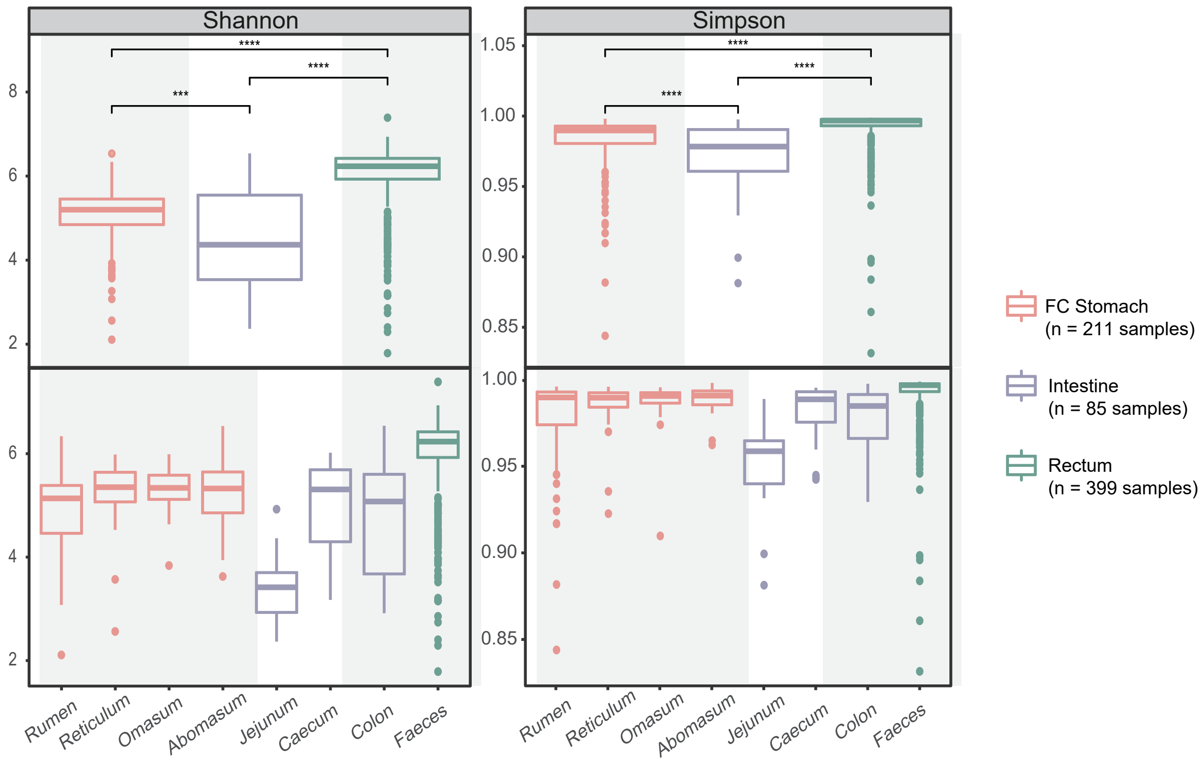

Supplementary Fig. 6: Comparison of the different alpha diversity among the digestive tract (DT).
Boxplots showing the alpha diversity measurements including Shannon (left panel) and Simpson (right panel) indexes of the DT sections (upper panel) and sites (lower panel). The different colours of boxes indicate the different sections of DT， which are FC stomach (orange, n= 211 samples), intestine (purple, n= 85 samples) and rectum (green, n= 399 samples).Pair-wise Wilcoxon Rank Sum Test was used to compare between the groups. Boxplots show median, 25th and 75th percentile, the solid line indicate the minima and maxima, and the points laying outside the whiskers of boxplots represent the outliers.
Level of significance: ns $P \geq 0.05$, * $P < 0.05$, ** $P < 0.01$, *** $P < 0.001$, **** $P < 0.0001$.

a

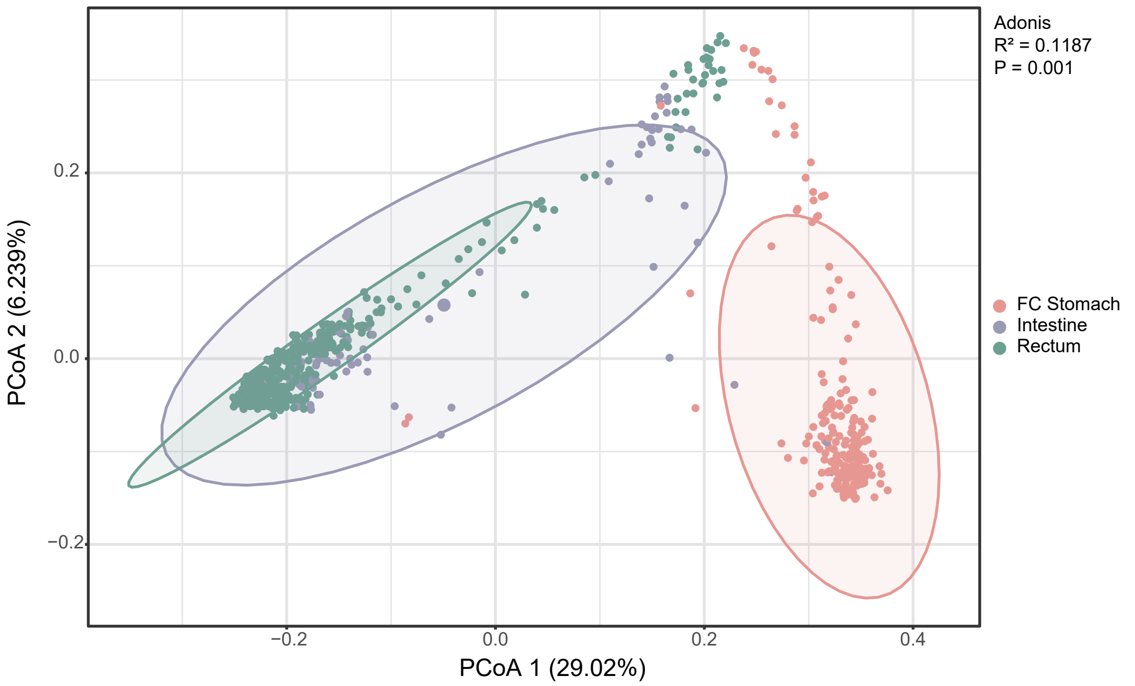

b

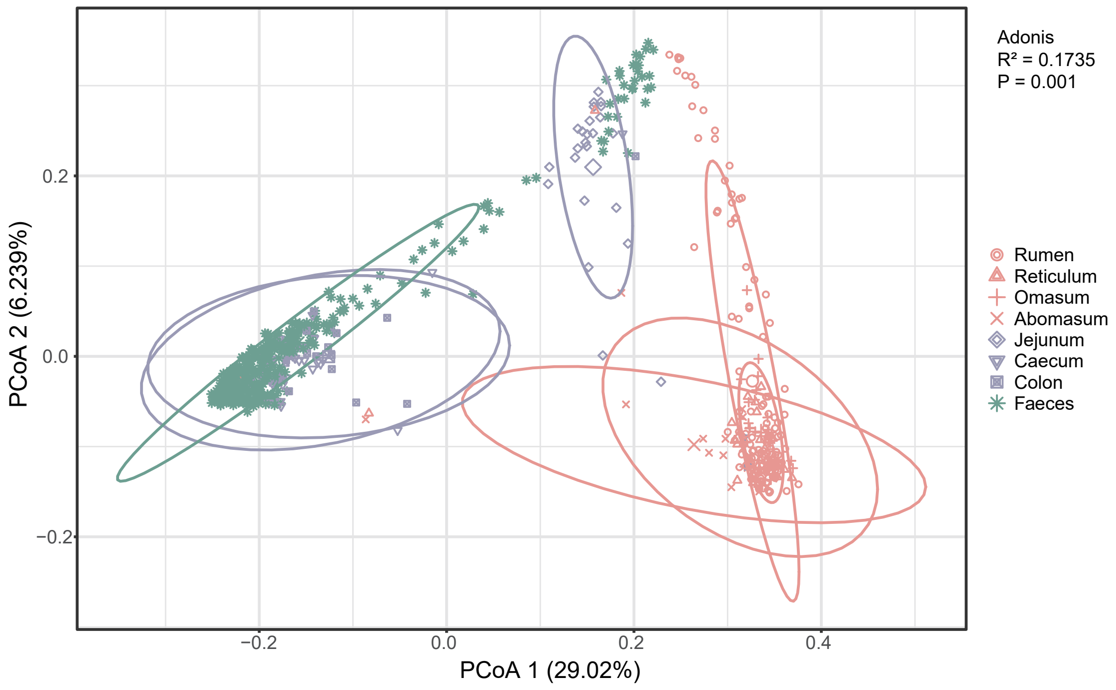

Supplementary Fig. 7: Principal coordinates analysis (PCoA) of the Bray-Curtis distances among different digestive tract (DT) sections and sits. a) Principal coordinates analysis (PCoA) analysis of Bray-Curtis distances among the samples for the three sections. The p-value and R2 were reported by the 'adonis' function from the 'vegan' package of R using a non-parametric MANOVA test (see Materials and Methods for details). b) Principal coordinates analysis (PCoA) analysis of Bray-Curtis distances among the samples for the eight DT sites.

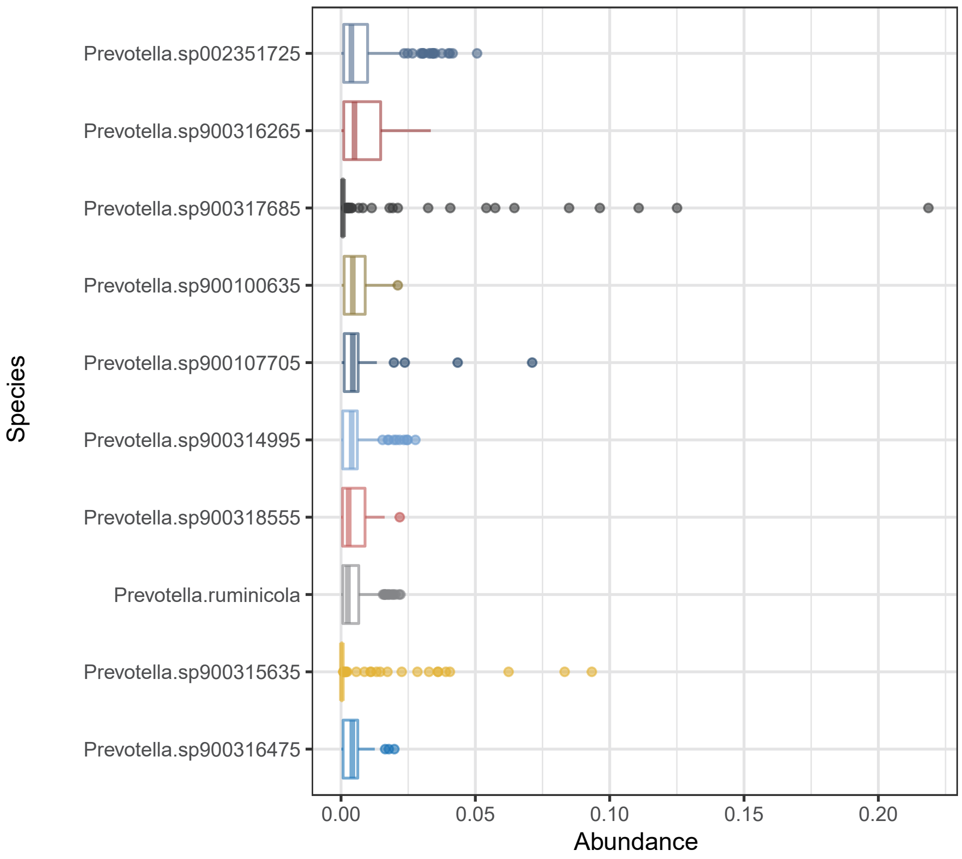

Supplementary Fig. 8: Boxplot of the Prevotella species with the highest abundance in rumen (n = 695 sample). X-axis indicate the relative abundance, and y-axis indicate top 10 abundances of Prevotella species. Boxplots show median, 25th and 75th percentile, the solid line indicate the minima and maxima, and the points laying outside the whiskers of boxplots represent the outliers.

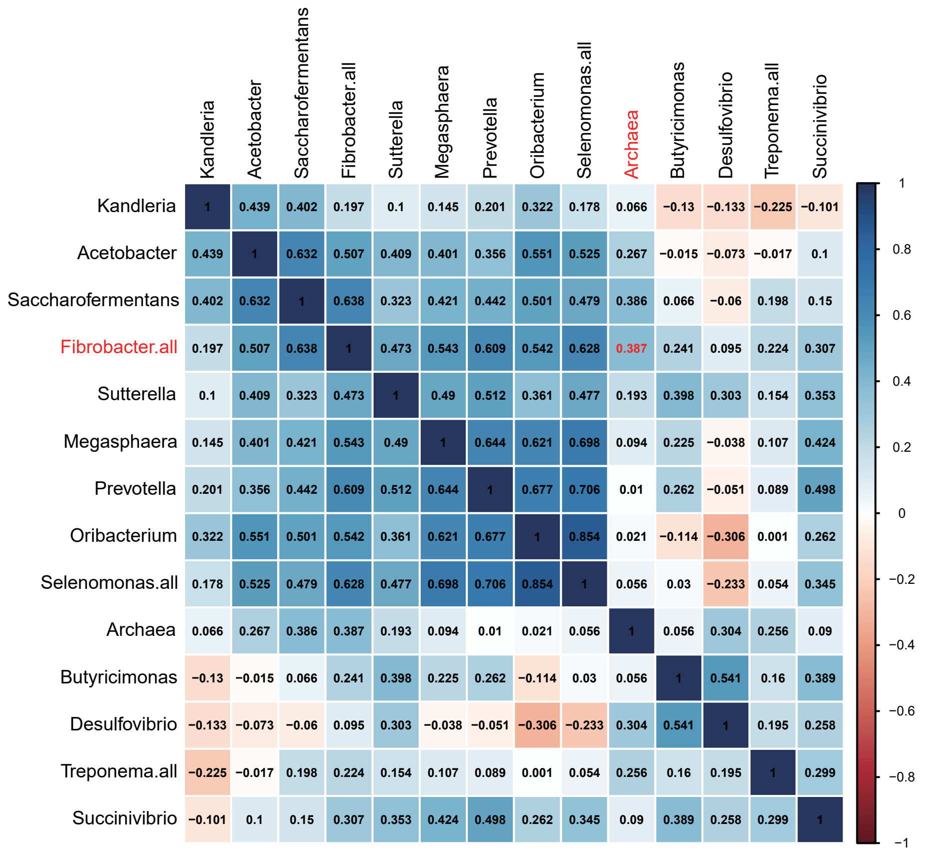

Supplementary Fig. 9: Correlation analysis among genera with known taxonomic classifications using their relative abundance in all DT samples. Correlation of all the genus and Archaea with functional information that are significantly related to the Archaea were calculated. Red indicate negative correlation, and blue indicate positive correlation. The number in each square showed the correlation coefficient of them.

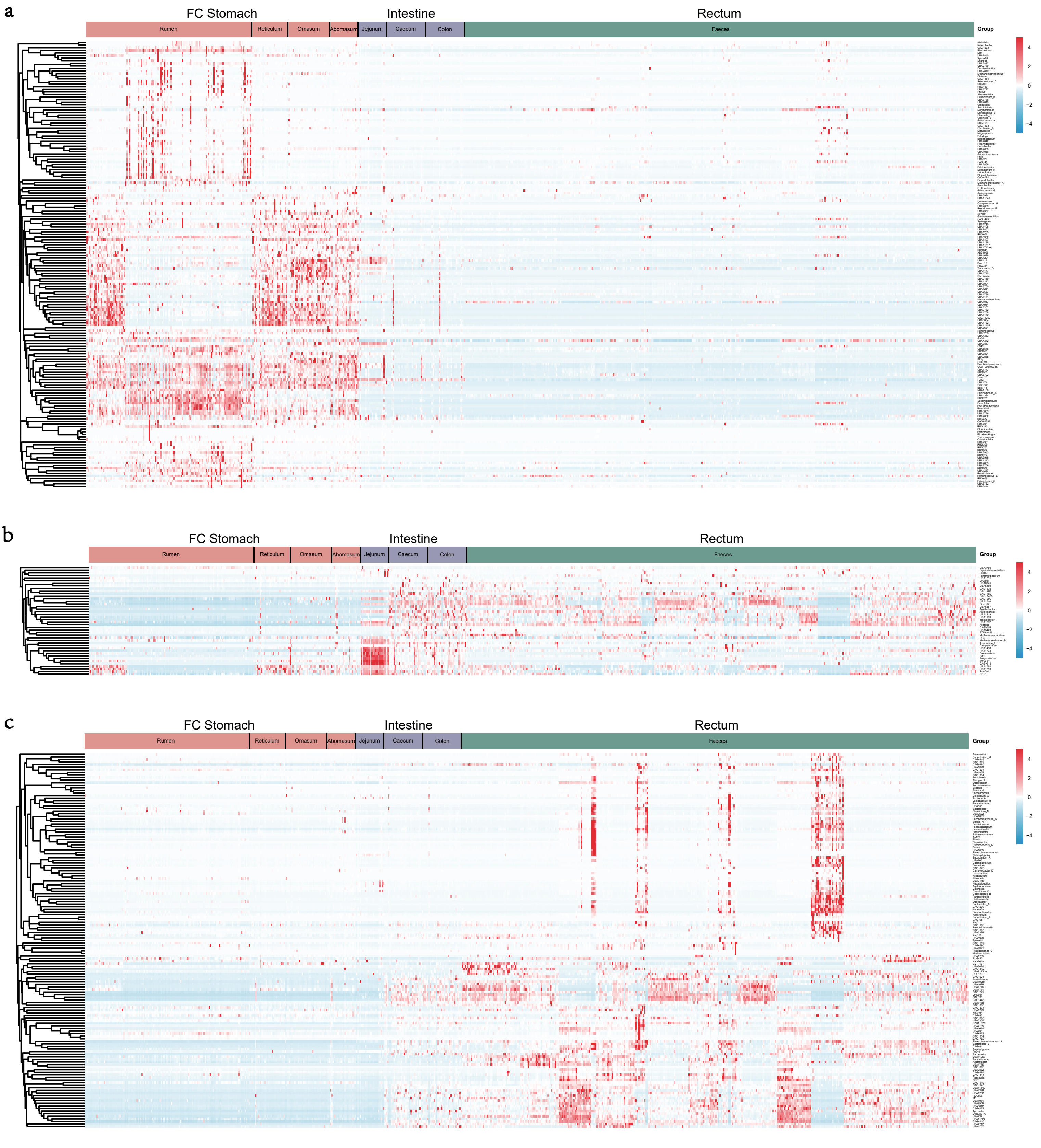

Supplementary Fig. 10: Relative abundance of all marker taxa in different sections along the digestive tract. The comparison of FC Stomachs, Intestines and Rectum at a) The relative abundance was higher in FC Stomachs, b) The relative abundance was higher in Intestines, and c) The relative abundance was higher in Rectum.

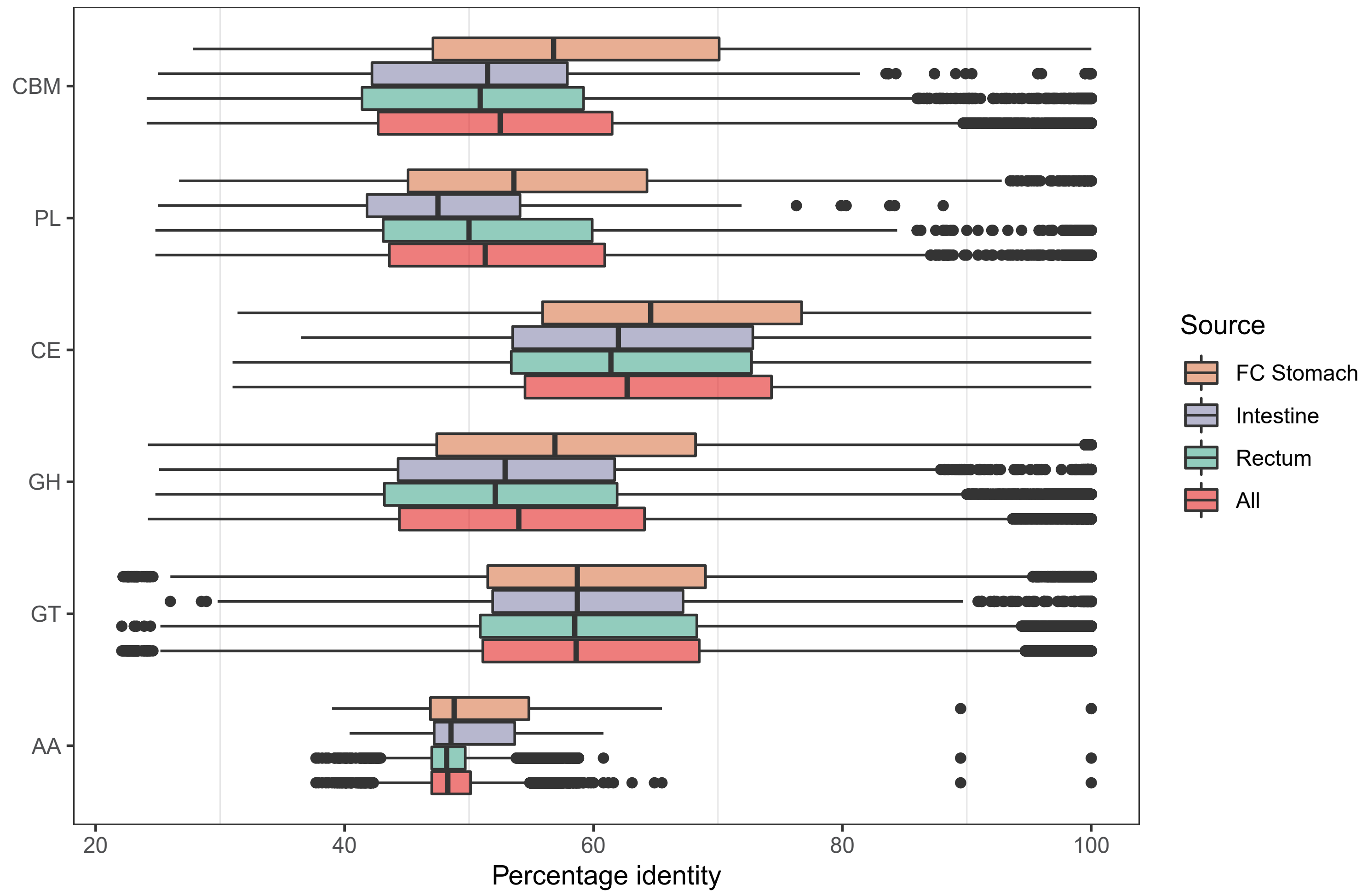

Supplementary Fig. 11: Maximum percent identity between predicted proteins from the MAGs and the CAZy database. X-axis indicate the percentage identity. Y -axis indicate six enzyme classes in CAZy database, glycoside hydrolases (GH, n = 76,224), glycosyl transferases (GT, n = 21,402), carbohydrate-binding modules (CBM, n = 11,645), carbohydrate esterases (CE, n = 3,804), polysaccharide lyases (PL, n = 1,879) and auxiliary activities (AA, n = 35). Boxplots show median, 25th and 75th percentile, the solid line indicate the minima and maxima, and the points laying outside the whiskers of boxplots represent the outliers.

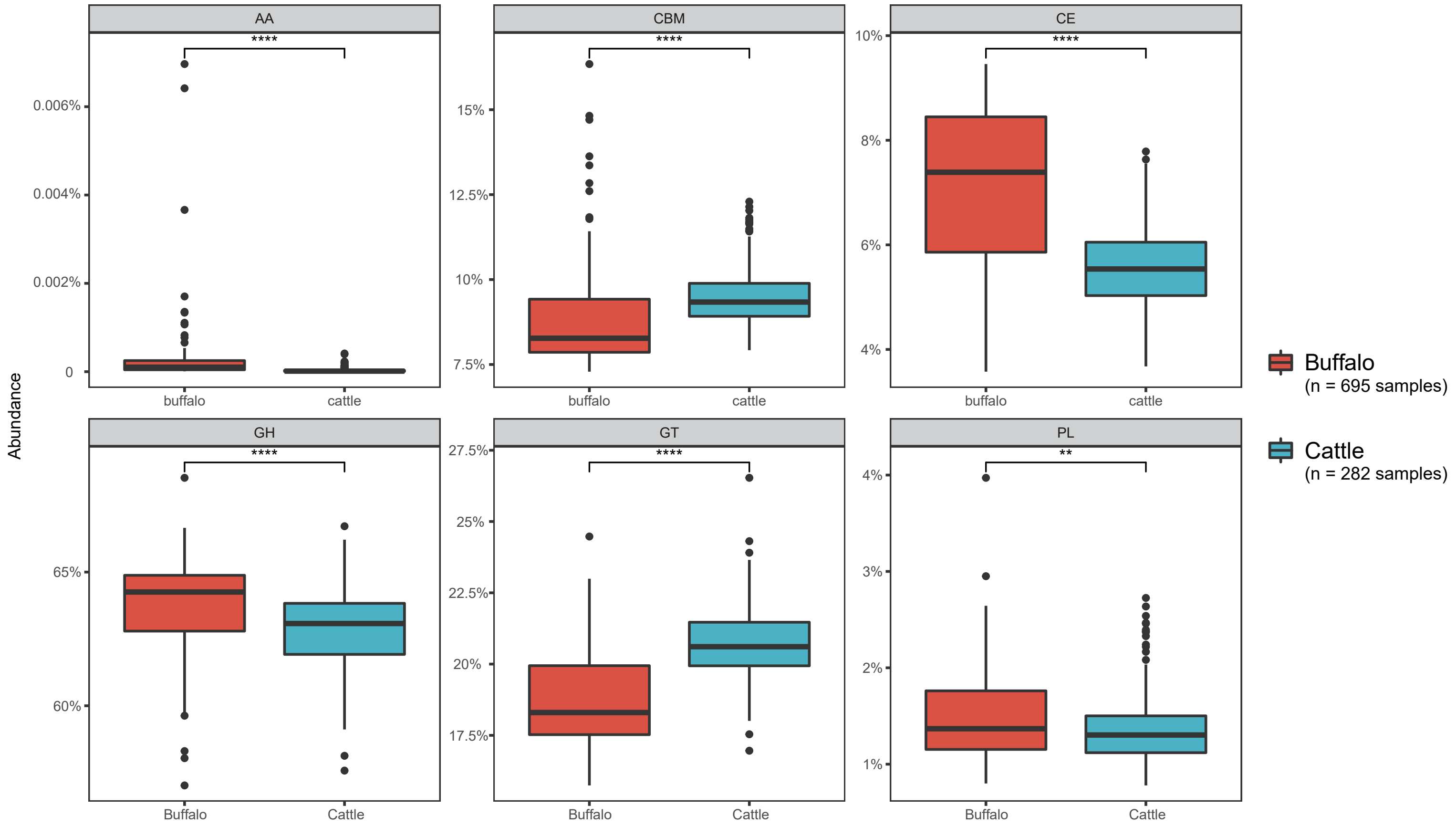

Supplementary Fig. 12: Comparison of the relative abundance of six CAZy families between buffalo and cattle based on all CAZy protein abundance values. X-axis indicate proteins belong to buffalo (red, n = 695 samples) and cattle (bule, n = 282 samples). Y-axis shows the relative abundances of protein families; GH, glycoside hydrolase; GT, glycosyl transferase; PL, polysaccharide lyase; CE, carbohydrate esterase; AA, auxiliary activities; CBM, carbohydrate-binding module. Boxplots show median, 25th and 75th percentile, the solid line indicate the minima and maxima, and the points laying outside the whiskers of boxplots represent the outliers. Pair-wise Wilcoxon Rank Sum Test was used to compare between the groups.
Level of significance: ns $P \geq 0.05$, * $P < 0.05$, ** $P < 0.01$, *** $P < 0.001$, **** $P < 0.0001$.

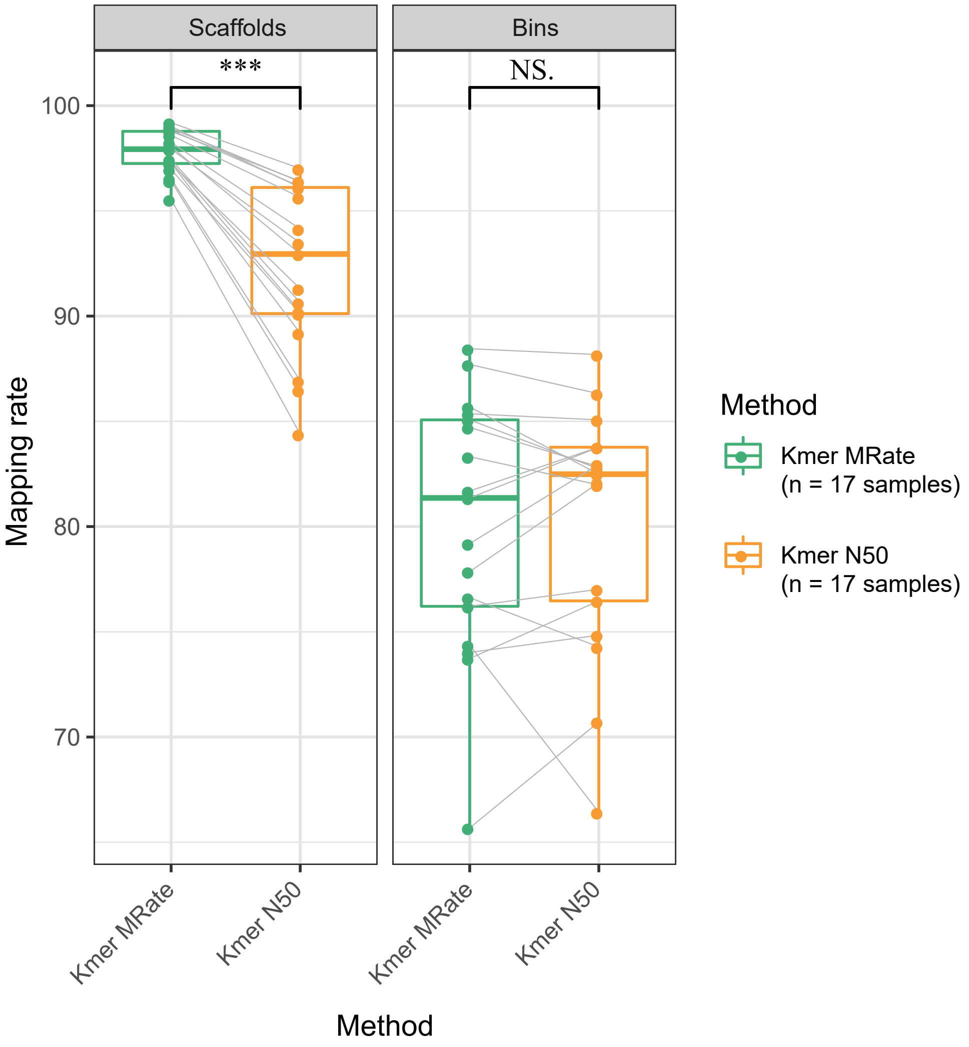

Supplementary Fig. 13: Effects of different assembly parameters on read mapping rates against the obtained scaffolds (left panel) and bins (right panel). Each dot represents a sample. Left panel: at the level of the scaffold, the mapping rates of the Kmer_MRate group were significantly higher than the Kmer_N50 group; Right panel: after binning, we found no significant difference at the bins level between the two groups.
Kmer_N50 (n = 17 samples): the Kmer used in our study to obtain the highest N50;
Kmer_MRate (n = 17 samples): the Kmer with the highest mapping rate (range 55 ~ 115， step increase 10). Pair-wise Wilcoxon Rank Sum Test was used to compare between the groups. Boxplots show median, 25th and 75th percentile,the solid line indicate the minima and maxima, and the points laying outside the whiskers of boxplots represent the outliers.
Level of significance: ns $P \geq 0.05$, * $P < 0.05$, ** $P < 0.01$, *** $P < 0.001$, **** $P < 0.0001$.

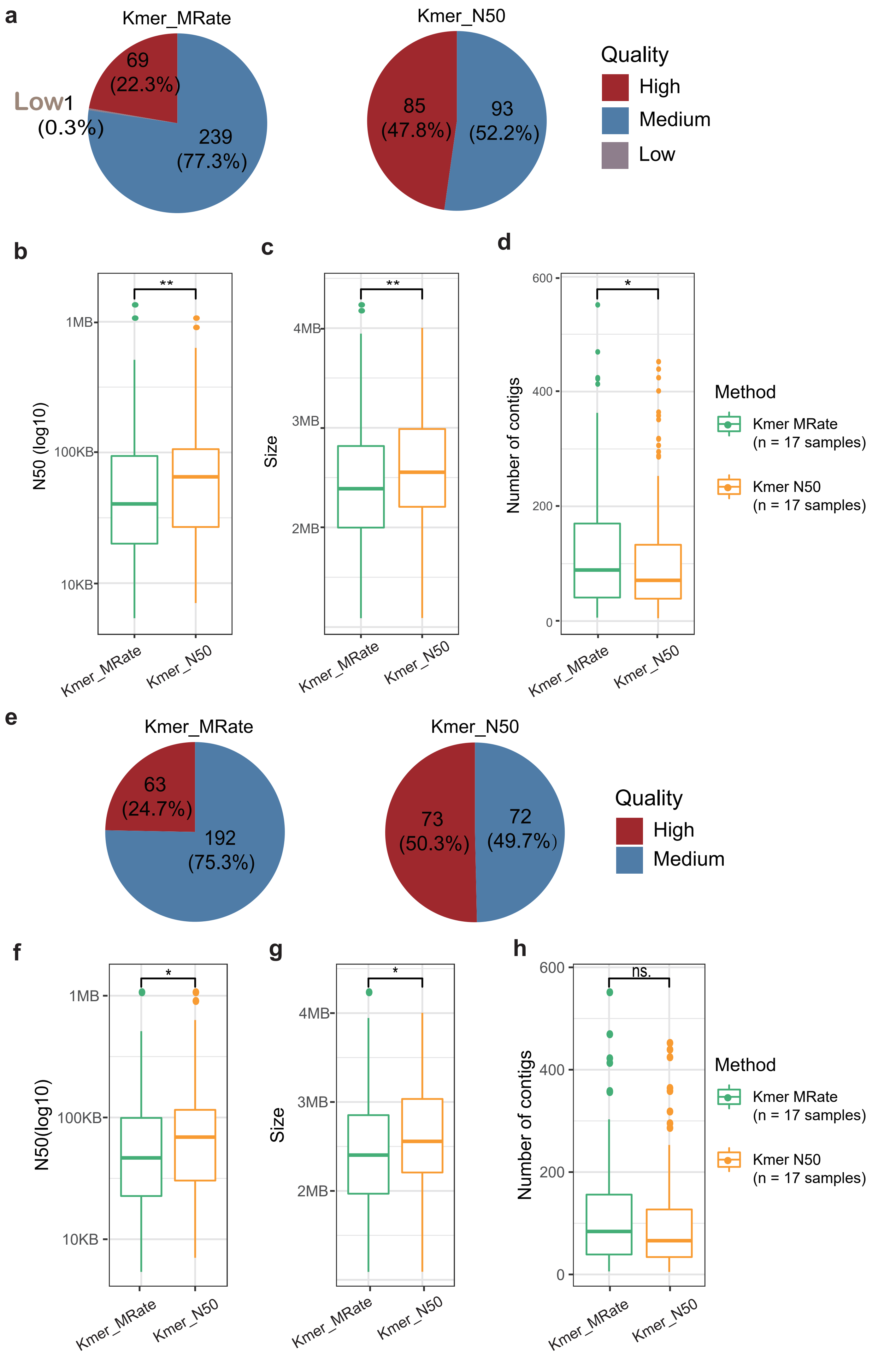

Supplementary Fig. 14: Quality assessment of the nonredundant MAGs generated using the Kmer_MRate and Kmer_N50 methods. a) Pie chart shows the numbers and relative proportions of the high-, medium- and low-quality MAGs generated using the Kmer_MRate and Kmer_N50 groups at the strain level. Here the "high-quality" MAGs are defined as those with ≥90% completeness and ≤5% contamination, and presence of the 23S, 16S, and 5S rRNA genes and at least 18 tRNAs. All other MAGs are >80% complete and ≤10% contaminated. Those in blue have a quality score ≥50 as defined by Parks et al 1, whereas those in grey have a quality score <50. b), c) and d) show the comparisons of N50, genome size and the number of contig per genome respectively for the MAGs assembled at strain level between the Kmer_MRate (n = 17 samples) and Kmer_N50 (n = 17 sampels) methods. Pair-wise Wilcoxon Rank Sum Test was used to compare between the groups. Boxplots show median, 25th and 75th percentile, the solid line indicate the minima and maxima, and the points laying outside the whiskers of boxplots represent the outliers. Level of significance: ns P ≥ 0.05, * P < 0.05, ** P < 0.01, *** P < 0.001, **** P < 0.0001. e) Pie chart shows the numbers and relative proportions of the high-, medium- and low-quality MAGs generated by the two at the species level. f), g) and h) show the comparisons of N50, genome size and the number of contig per genome respectively for the MAGs assembled at species level between the two methods. Pair-wise Wilcoxon Rank Sum Test was used to compare between the groups. Boxplots show median, 25th and 75th percentile, the solid line indicate the minima and maxima, and the points laying outside the whiskers of boxplots represent the outliers.
Level of significance: ns P ≥ 0.05, * P < 0.05, ** P < 0.01, *** P < 0.001, **** P < 0.0001.

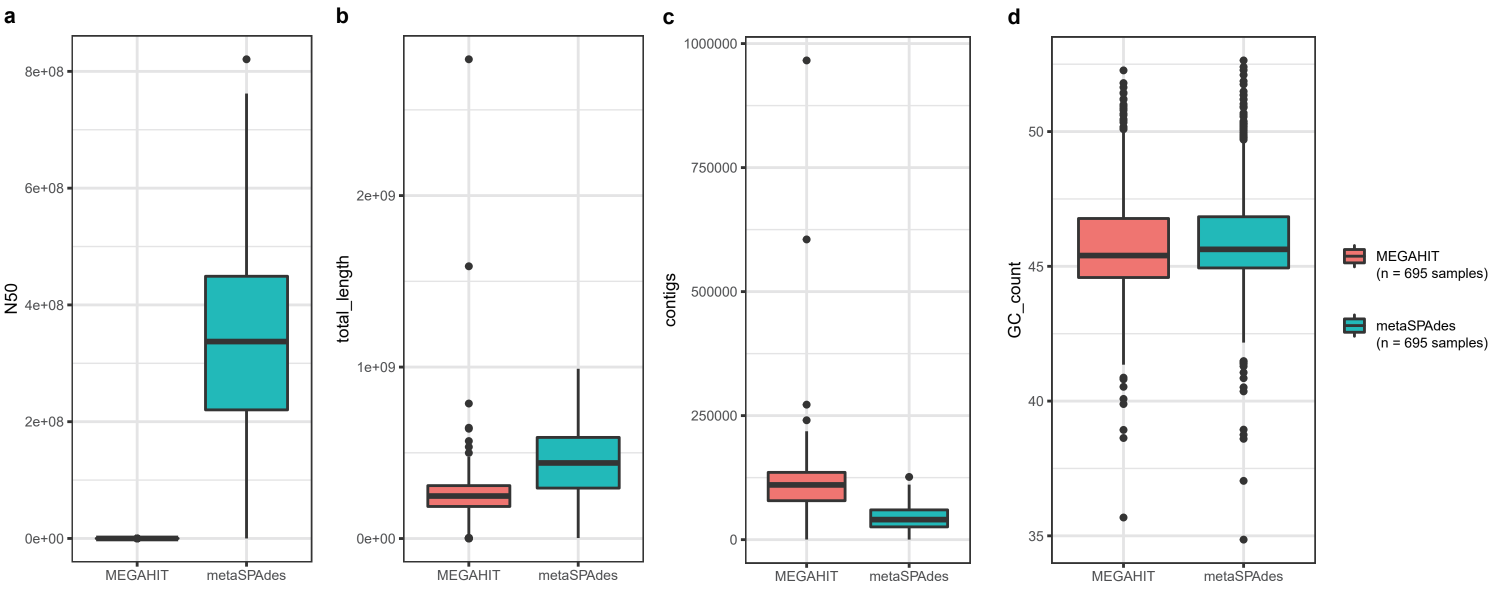

Supplementary Fig. 15: Boxplot comparison of the assembly results between metaSPAdes (blue) and MEGAHIT (red). The comparison of metaSPAdes (n = 695 samples) and MEGAHIT (n = 695 samples) at a) N50, b) total length, c) contigs numbers, and d) GC counts level. Boxplots show median, 25th and 75th percentile, the solid line indicate the minima and maxima, and the points laying outside the whiskers of boxplots represent the outliers.
