# Supplementary material for: The microbiome of the buffalo digestive tract
Source: Nat Commun. 2022 Feb 10;13:823. doi: 10.1038/s41467-022-28402-9 (PMC8831627; doi:10.1038/s41467-022-28402-9)
Supplement: Supplementary file 3 — Description of Additional Supplementary Files [file 41467_2022_28402_MOESM3_ESM.docx]

**Description of Additional Supplementary Files**

**File Name:** Supplementary Data 1

**Description:** Summary of sample collection. Numbers of samples from different sampling locations.

**File Name:** Supplementary Data 2

**Description:** Quality and taxonomic information of 4960 MAGs.

**File Name:** Supplementary Data 3

**Description:** Correlation indexes between Archaea and other genera.

**File Name:** Supplementary Data 4

**Description:** Differences in the relative abundance of different genera according to LEfSe.

**File Name:** Supplementary Data 5

**Description:** Sample sequencing information.

**File Name:** Supplementary Data 6

**Description:** Qualigy control information of sequenced samples.

**File Name:** Supplementary Data 7

**Description:** Differences in mapping rates between Kmer_N50 and Kmer_MRate.

**File Name:** Supplementary Data 8

**Description:** Dereplication (dRep) results using different ‘-nc’ options.

**File Name:** Supplementary Data 9

**Description:** Taxonomic annotation of the different bins.
