# Supplementary material for: The microbiome of the buffalo digestive tract
Source: Nat Commun. 2022 Feb 10;13:823. doi: 10.1038/s41467-022-28402-9 (PMC8831627; doi:10.1038/s41467-022-28402-9)
Supplement: Supplementary file 13 — Reporting Summary [file 41467_2022_28402_MOESM13_ESM.pdf]

nature research

Corresponding author(s): Qingyou Liu

Last updated by author(s): Dec 23, 2021

Reporting Summary

Nature Research wishes to improve the reproducibility of the work that we publish. This form provides structure for consistency and transparency in reporting. For further information on Nature Research policies, see our Editorial Policies and the Editorial Policy Checklist.

Statistics

For all statistical analyses, confirm that the following items are present in the figure legend, table legend, main text, or Methods section.

n/a	Confirmed	
☒	☐	The exact sample size (n) for each experimental group/condition, given as a discrete number and unit of measurement
☒	☐	A statement on whether measurements were taken from distinct samples or whether the same sample was measured repeatedly
☒	☐	The statistical test(s) used AND whether they are one- or two-sided<br>*Only common tests should be described solely by name; describe more complex techniques in the Methods section.*
☒	☐	A description of all covariates tested
☐	☒	A description of any assumptions or corrections, such as tests of normality and adjustment for multiple comparisons
☐	☒	A full description of the statistical parameters including central tendency (e.g. means) or other basic estimates (e.g. regression coefficient) AND variation (e.g. standard deviation) or associated estimates of uncertainty (e.g. confidence intervals)
☒	☐	For null hypothesis testing, the test statistic (e.g. F, t, r) with confidence intervals, effect sizes, degrees of freedom and P value noted<br>*Give P values as exact values whenever suitable.*
☒	☐	For Bayesian analysis, information on the choice of priors and Markov chain Monte Carlo settings
☐	☒	For hierarchical and complex designs, identification of the appropriate level for tests and full reporting of outcomes
☐	☒	Estimates of effect sizes (e.g. Cohen's d, Pearson's r), indicating how they were calculated

Our web collection on statistics for biologists contains articles on many of the points above.

Software and code

Policy information about availability of computer code

Data collection	No software was used in data collection.
Data analysis	Standalone tools include Trimmomatic (v.0.35), bowtie2 (v.2.3.3), MEGAHIT (v.1.2.8), metaSPAdes (v.3.13.0), BWA-MEM (v.0.7.15), MetaBAT2 (2.12.1), Samtools (v.1.8), dRep (v.2.3.2), BEDTools (v.2.27.1), Prodigal (v2.6.3), CD-HIT (v.4.8.1), eggnog-mapper (v.4.5), dbCAN2 (v.2.0), GTDB-TK (v.1.4.1) and GraPhlAn (v.1.1.3) were used. In addition, in-house R (v.4.0) scripts were used for data processing, analysis, statistics and data visualisation (https://github.com/fengtong-bio/MEABDT).

For manuscripts utilizing custom algorithms or software that are central to the research but not yet described in published literature, software must be made available to editors and reviewers. We strongly encourage code deposition in a community repository (e.g. GitHub). See the Nature Research guidelines for submitting code & software for further information.

Data

Policy information about availability of data

All manuscripts must include a data availability statement. This statement should provide the following information, where applicable:

- Accession codes, unique identifiers, or web links for publicly available datasets
- A list of figures that have associated raw data
- A description of any restrictions on data availability

The raw sequencing data used in this study are available in the NCBI SRA database under accession code PRJNA656389 (https://www.ncbi.nlm.nih.gov/bioproject/PRJNA656389). The 4,960 strain-level MAGs data used in this study are available in the Figshare database under accession code 17000302 (https://figshare.com/articles/dataset/Buffalo_digestive_tract_metagenome_MAGs/17000302). The 3,255 species-level MAGs data used in this study are available in the Figshare database under accession code 17081366 (https://figshare.com/articles/dataset/Species-level_3255_Buffalo_digestive_tract_metagenome_MAGs/17081366). Source data are provided with this paper and these data have been made public.

Field-specific reporting

Please select the one below that is the best fit for your research. If you are not sure, read the appropriate sections before making your selection.

☒ Life sciences ☐ Behavioural & social sciences ☐ Ecological, evolutionary & environmental sciences

For a reference copy of the document with all sections, see nature.com/documents/nr-reporting-summary-flat.pdf

Life sciences study design

All studies must disclose on these points even when the disclosure is negative.

Sample size	A total of 695 samples were collected for metagenome sequencing. To ensure the diversity of the samples, they were collected from three breeds (153, 430, 112 samples from river, swamp, and hybrid buffaloes), six regions (Guangxi, Henan, Anhui, Yunnan, Hainan, Hubei from China), two sexes (females and males respectively) and two developmental stages (Adult buffaloes and calves). Out of the total 695 samples, 497 were taken from live buffalo, including 98 rumen samples collected by stomach tube and 399 fecal samples collected by disposable soft long arm gloves from the rectum. The remaining 198 samples were all from slaughtered buffalo, including 31 rumen samples, 28 reticulum samples, 32 omasum samples, 22 abomasum samples, 24 jejunum samples, 31 cecum samples, and 30 colon samples . Rarefaction curves of assembled genomes (MAGs) shows that the number of MAGs has reached saturation, so the sample size is sufficient.
Data exclusions	No data were excluded from the analysis.
Replication	All the results of this study are reproducible and reproducible. According to the original data and workflow provided by us, the results of our analysis can be reproduced.
Randomization	In order to ensure the accuracy of the research, all metagenomic samples used for sequencing are randomly selected when they meet the requirements.
Blinding	The sample collection object of this study is a buffalo samples, so blinding was not relevant in the study.

Reporting for specific materials, systems and methods

We require information from authors about some types of materials, experimental systems and methods used in many studies. Here, indicate whether each material, system or method listed is relevant to your study. If you are not sure if a list item applies to your research, read the appropriate section before selecting a response.

Materials & experimental systems

n/a	Involved in the study	
☒	☐	Antibodies
☒	☐	Eukaryotic cell lines
☒	☐	Palaeontology and archaeology
☐	☒	Animals and other organisms
☒	☐	Human research participants
☒	☐	Clinical data
☒	☐	Dual use research of concern

Methods

n/a	Involved in the study	
☒	☐	ChIP-seq
☒	☐	Flow cytometry
☒	☐	MRI-based neuroimaging

Animals and other organisms

Policy information about studies involving animals; ARRIVE guidelines recommended for reporting animal research

Laboratory animals	No.
Wild animals	The buffalo samples in this study do not involve wild animals.
Field-collected samples	All samples were collected in outdoor environment with room temperature and humidity.
Ethics oversight	The investigation was approved by the Experimental Animal Ethics Committee, College of Animal Science and Technology, Guangxi University, under reference number Gxu-2021-010.

Note that full information on the approval of the study protocol must also be provided in the manuscript.
